# Leaders’ Windows of Tolerance for Affect Arousal—and Their Effects on Political Decision-making During COVID-19

**DOI:** 10.3389/fpsyg.2021.749715

**Published:** 2021-10-26

**Authors:** Kelsey L. Larsen, Elizabeth A. Stanley

**Affiliations:** ^1^School of Politics, Security, and International Affairs, University of Central Florida, Orlando, FL, United States; ^2^School of Foreign Service, Georgetown University, Washington, DC, United States

**Keywords:** leaders, emotion, stress, decision-making, self-regulatory capacity, Donald Trump, Jacinda Ardern, COVID-19

## Abstract

The recent ‘affect revolution’ in strategic decision-making research has placed greater emphasis on the role of stress and emotions in decision-making, with new theorizing to highlight how leader decisions often differ from rational choice expectations. However, while existing theories add to our understanding of the interplay between affect and cognition, they have not yet explained why affect drives decisions in some situations and not others. Undertheorized connections between leaders’ neurobiological windows of tolerance to affect arousal and their self-regulatory capacity—their capacity to regulate stress and emotions so that these phenomena do not drive resulting decisions—may hold the key to explaining this variation in affect’s influence on decision-making. Furthermore, this article considers how leaders’ windows of tolerance have unique ripple effects in their social environments, thereby affecting their groups’ *collective* window of tolerance. While regulated leaders can convey a calming and creative influence in their organizations that helps the group access strategic decision-making, dysregulated leaders are likely to convey stress and emotion contagion—which may erode the group’s ability to cooperate, adapt, and learn. It illustrates this argument using evidence from the upper echelons of governmental decision-making, comparing New Zealand Prime Minister Jacinda Ardern’s and US President Donald Trump’s responses to the coronavirus pandemic in their respective nations. It concludes by offering hypotheses for testing the argument in future empirical research.

## Introduction

As the coronavirus (COVID-19) pandemic spread across the globe in early 2020, New Zealand Prime Minister Jacinda Ardern turned to scientists. She solicited input from leading experts, worked with her cabinet to forecast New Zealand’s future, and communicated directly with the public. In briefings and Facebook Live discussions, Ardern promoted a clear-eyed strategy of strength and kindness, bluntly assessing the threat the virus posed and introducing the required lockdown measures. Cabinet officials, several opposition leaders, and almost all New Zealanders bought into her approach. Along with her ‘team of five million,’ Ardern led one of the most successful fights against COVID-19 to date, with just 27 total deaths and an average daily case rate below 20 (World Health Organization, 2021a).

US President Donald Trump took a different approach. He inconsistently called upon and dismissed the advice of experts, rejected the perspectives of anyone deemed disloyal, and used Twitter and press briefings to deliver erratic and impromptu messaging to the US public. His inner circle was plagued by toxic in-fighting, while Americans were confused and distrusting of official guidelines. With more than 663,000 COVID-19 deaths to date, the United States continues to have an average daily case rate of more than 156,000 (World Health Organization, 2021b).

What explains this difference in how these two leaders chose to respond to the shared experience of the COVID-19 pandemic? Certainly, these cases differ in physical context: New Zealand is a small island nation with greater ease for enacting a quarantine, while the more populous United States has many ports of entry and is a hub for international travel. Ardern’s and Trump’s responses could partially be explained by these differing contexts, as well as by varying political institutions (parliamentary vs. presidential democracy) or individual variables, such as personality traits and gender. Nonetheless, recent advances in the decision-making literature would suggest that affect—including stress arousal, emotions, and moods (Gross, 2015)—should have had equal impact on the two leaders’ decision-making, given that they faced the same complex threat. Why did it not? We suggest that at least some of their difference can be attributed to overlooked elements in the decision-making literature: the leaders’ neurobiological windows of tolerance to affect arousal and their self-regulatory capacity.

This article proceeds as follows. The first section reviews existing theories about leaders’ affect arousal, including its direct effects on their strategic decision-making and its indirect effects on their groups. The second section offers a theory designed to explain the intersection of emotion and stress—and how they together influence both individual and collective decision-making and behavior. We introduce the concept of leaders’ neurobiological windows of tolerance for affect arousal and explore how this window can directly affect leaders’ capacity to make effective decisions. We also consider how leaders’ windows have unique ripple effects in their social environments, thereby indirectly affecting their groups’ *collective* window of tolerance. The third section offers a formal illustration of the argument, comparing Ardern’s and Trump’s responses to the COVID-19 pandemic, to show how leaders’ windows can affect their decision-making and their group’s collective performance. The final section develops specific hypotheses and offers recommendations for systematically testing this new idea in empirical research.

## Leader Affect Arousal

Over the last two decades, there has been growing interest in the intersection of emotions and government (e.g., Crawford, 2000; Redlawsk, 2006; Neuman et al., 2007; Groenendyk, 2011; Halperin et al., 2011), especially regarding how affect conditions political decision-makers’ strategic choices (e.g., Bar-Joseph and McDermott, 2008; Dolan, 2016; Renshon et al., 2017; Stanley, 2018). Drawing on recent advances in social and organizational psychology—including psychological constructionism (Barrett, 2009, 2017), cognitive appraisal theory (Smith and Ellsworth, 1985; Frijda, 1986; Moors, 2013), basic emotion theory (Ekman, 1972; Ekman and Cordaro, 2011), and the somatic marker hypothesis (Damasio, 1994; Verweij and Damasio, 2018)—this literature examines how leader affect complements (and often directly shapes) cognitive processing, with varying effects on the leader’s own decision-making and their subordinates’ decisions and behaviors. Yet, this research generally fails to consider how leader self-regulatory capacity intersects with leader affect.

While the various theoretical perspectives have differing views about affect and its relationship with cognition, this field takes as a common foundation that affective experiences can work in tandem with cognition to shape how individuals think, act, and make decisions—with affect shaping decisions and behavior *via* goal motivation, the content of thought, and the depth of thought, among other functions (Druckman and McDermott, 2008; Renshon and Lerner, 2011; Lerner et al., 2015; Cristofaro, 2019, 2020). Affect shapes individuals’ cognitive processing by influencing “what kind of information people recall, attend to, select, interpret, and learn” (Forgas and George, 2001, p. 8)—with concomitant effects on attentiveness, memory retrieval, information searches, and risk/utility calculations (for a review, see Cristofaro, 2019, 2020; see also Forgas and George, 2001; Pham, 2007; Seo and Barrett, 2007; Angie et al., 2011). Often, though not universally, ‘positive’ affect (i.e., positive moods; emotions such as joy and contentment) is argued to promote top-down, flexible processing rooted in approach/continue behaviors—in turn facilitating action tendencies related to creativity, motivation, cooperation, resilience, open attention, and a reliance on existing schema (Isen et al., 1987; George, 1991; Bless, 2000; Fredrickson, 2001; Seo et al., 2004; Amabile et al., 2005; Fredrickson and Branigan, 2005). In contrast, ‘negative’ affect (i.e., negative moods; stress; emotions such as anxiety, sadness, and anger) is argued to motivate avoidance—in turn facilitating pessimism, antagonism, defensiveness, resistance, enhanced selective attention, and cognitive rigidity in decision-making (Sinclair and Mark, 1992; Conway and Giannopoulos, 1993; Elsbach and Barr, 1999; Finucane, 2011; Robinson et al., 2011).

Of course, whether affect enhances or maladaptively impedes leaders’ strategic decision-making is highly context-specific across both space and time, as the leadership literature shows (Humphrey et al., 2016; Cristofaro, 2019). For instance, under significant time constraints, happy managers have been shown to be *less* creative and make worse decisions, while sad managers make better ones (Treffers et al., 2020). In related research, decision-makers experiencing happiness and anger may have difficulties processing decision-relevant information, while those experiencing moderate fear are more likely to make rationally strategic choices (Coget et al., 2011; Bachkirov, 2015). Likewise, in political environments, recent evidence finds that individual citizens experiencing anger are motivated toward political participation (Valentino et al., 2011) and support for aggressive security policies, including war declarations (Lerner et al., 2003; Huddy et al., 2005; Halperin, 2011). In contrast, anxiety has been shown to motivate information-seeking about political candidates (Redlawsk et al., 2007; Valentino et al., 2008), while sadness has been shown to motivate depolarization of ideological schema (Gur et al., 2021). Other research illuminates the varied effects of positive affect—with joy driving political leaders to devalue risk perceptions and embrace objectively riskier strategies, and contentment driving leaders to resist strategic change (Dolan, 2016).

One important body of research focuses on various forms of physiological arousal, finding relationships between measures such as electrodermal activity and political preferences and behaviors [though there are inconsistencies in replications; see Smith and Warren (2020) for a review]. Other experimental research about bargaining behavior demonstrates that affective arousal—regardless of valence—inhibits decision-makers’ deliberative processes and short-circuits their ability to make optimal cognitive choices (Renshon et al., 2017). These experimental findings corroborate empirical evidence that increased physiological and emotional arousal undermines leaders’ crisis decision-making (Bar-Joseph and McDermott, 2008) and exacerbates other war-lengthening dynamics, helping to explain why longer wars are harder to end (Stanley, 2018).

In addition to its direct effects on the leader’s own decision-making, leader affect also indirectly influences the perceptions, decisions, and behaviors of their subordinates. Several models explain such contagion. The first model follows the logic of affect-as-information—wherein followers glean information about a situation from their leaders’ emotions and moods, which they then use to make cognitively-informed assessments (Schwarz and Clore, 1983; Petty et al., 1994; Van Kleef, 2008). The second model follows the logic of appraisal theory, wherein followers’ own affective responses to their leaders’ emotions provide information for their cognitive assessments (Parkinson, 2011). A third model posits contagion as an automatic, unconscious, and unintentional transference of affect between leaders and followers (Hatfield et al., 1994). Leader affect evokes similar physiological processes in followers, such that they automatically mimic their leader’s verbal and non-verbal cues and converge with their emotions (Neumann and Strack, 2000; Bono and Ilies, 2006; Johnson, 2008; Spoor and Kelly, 2009; Clarkson et al., 2020).

Multiple tests of these mechanisms confirm emotion contagion from the leader can impact followers’ individual and collective decision-making and behavior (for reviews, see Cristofaro, 2019, 2020; see also Newcombe and Ashkanasy, 2002; Gaddis et al., 2004). For instance, leaders in positive moods engender followers with positive and/or less negative moods, while leaders in negative moods engender followers with negative and/or less positive moods (George, 1995; Sy et al., 2005). These mood shifts then impact followers’ behavior: Those who received positive contagion exhibited greater effort, coordination, and creativity, improved decision-making, and better overall performance (Sy et al., 2005; Bono and Ilies, 2006; Johnson, 2009; Visser et al., 2013; however, for an alternative perspective, see Barasch et al., 2016). In contrast followers who received negative contagion demonstrated varied behaviors, from less willingness to perform, to greater reliance on analytical approaches and increased effort (Johnson, 2009; Visser et al., 2013; Koning and Van Kleef, 2015; Lindebaum et al., 2016). At the same time, affective contagion has been shown to shape group decision-making dynamics—although these effects may be moderated by the affective context (e.g., group norms around affect; for a review, see Cristofaro, 2019).

In the political context, leaders’ positive and negative emotional displays have been shown to be “easily recognized and function as effective information processing cues” for their followers, who then alter their political attitudes and behaviors (Masters et al., 1991, p. 378; see also Rosenberg et al., 1986; Bucy and Bradley, 2004; Stewart and Svetieva, 2021). Other empirical evidence has supported the automatic contagion model, finding that Americans shared arousal reactions congruent with a president’s facial displays of emotion—irrespective of their attitudes toward that president (Lanzetta et al., 1985; McHugo et al., 1985; Masters, 2001).

Beyond these direct and indirect effects of leader affect arousal, research has explored some factors that mediate how emotions are experienced by leaders and caught by followers—thereby influencing decision-making for both. These factors include personality traits (Bartone, 2006; Van Kleef et al., 2010); gender (Lewis, 2000; Newcombe and Ashkanasy, 2002); the processes of sensegiving and sensemaking (for a review, see Cristofaro, 2021); and epistemic motivation (Sy et al., 2005; Van Kleef et al., 2009). Yet, this research generally fails to consider the mediating factor of leader self-regulatory capacity.

Affect regulation, like the umbrella term of affect itself, encompasses varying efforts to influence affect arousal (Westen, 1994; Gross, 2015). Leaders experience individual differences in their capacity to regulate their affect, which mediates how affect influences their own decision-making as well as how they might spread their affect arousal to followers. For example, one form of affect regulation—emotion regulation (ER)—refers to the processes that individuals use to influence which, how, and when emotions are experienced, and has five unique strategies (see Gross, 1998, 2015; Suri and Gross, 2016). Of these five ER approaches, which strategies are available (and selected) depend on the context, emotional intensity, and the individual’s executive functioning capacity (Heatherton and Wagner, 2011; Hofmann et al., 2012; Suri and Gross, 2016).

Alternatively, the ability to regulate stress arousal happens through allostasis, which relies on interactions between the brain, endocrine system, immune system, and autonomic nervous system to vary internal conditions—such that they galvanize the appropriate energy and focus for coping well before, during, and after a challenge (McEwen and Lasley, 2002; Stanley, 2019). When allostasis is functioning appropriately, stress arousal is an immediate response intended to handle change or crisis, followed by recovery and a return to baseline equilibrium. When an individual experiences chronic stress, however, they do not complete recovery; instead, they remain in an activated state. Over time, the internal systems involved with allostasis become dysregulated, in the process building allostatic load (Stanley, 2019).

Thus, all leaders’ capacity to use ER and allostasis to regulate their affect—to ensure that stress and emotions do not drive their own decision-making and, in turn, their followers’ decision-making—may not be equal. Like other leader-specific factors explored in the literature, leader self-regulatory capacity likely mediates how and when affect influences strategic decision-making, as some recent co-evolutionary organizational research has suggested (for a review, see Cristofaro, 2019; see also Ashkanasy, 2003; Fink and Yolles, 2015). Yet, most literature about affect and cognition in decision-making fails to engage with this idea—instead treating affect as a relatively constant influence on cognition and decision-making. To address this gap, we present an argument that incorporates leaders’ neurobiological windows of tolerance to affect arousal and their self-regulatory capacity as mediating factors that shape the interrelationships between affect, cognition, and strategic decision-making.

## The Mechanisms of Leader Self-Regulatory Capacity: Neurobiological Windows of Tolerance

In this section, we explore how leaders’ self-regulatory capacity can impact their organizations, in at least two ways. First, we review the logic of dual systems decision-making, introduce the concept of leaders’ neurobiological windows of tolerance for affect arousal, and survey how this window can directly affect leaders’ own decision-making. Then, we consider how leaders’ windows can indirectly affect the collective performance of their groups.

Leaders make decisions drawing upon two networked systems, System 1 and 2. Most applications of the dual systems decision-making model in the organizational literature have focused on System 1 guiding emotion and intuition, while System 2 steers cognition (for a review, see Cristofaro, 2020). Approaching this model from the stress and trauma neurobiological perspective, however, System 1 (“thinking fast”; Kahneman, 2011) is designed to deploy *neuroception*: an unconscious process that quickly scans both the inner and outer world for opportunities and threats (Porges, 2011; Stanley, 2019). As a result of this process, System 1 activates neurotransmitters and stress hormones, which then produce physical sensations and emotional cues associated with approaching opportunities or avoiding threats. System 1 is guided by these fast, automatic, and unconscious assessments, rather than conscious thought (Stanovich and West, 2000; Kahneman, 2003), and cultivates *implicit memory* by unconsciously interpreting and generalizing from every experience. System 1 implicit learning occurs predominantly in the amygdala during all levels of affective arousal, with the greatest learning occurring at high arousal levels (Bremner, 2002; Sapolsky, 2004; Scaer, 2005; Stanley, 2019).

System 2 (“thinking slow”; Kahneman, 2011) is the slower and more effortful pathway characterized by conscious thought. System 2 includes executive functioning, which allows us “to focus, pay attention, and recall task-relevant information, while holding distractions at bay” (Stanley, 2019, p. 99). System 2 also enables top-down conscious control to modify or override System 1’s bottom-up nonconscious assessments (Stanovich and West, 2000). System 2 employs *explicit memory*, which can be intentionally accessed to situate information in space and time—and which supports System 2 explicit learning, located primarily in the hippocampus (Scaer, 2005; Stanley, 2019).

An individual’s situational ability to access System 2 processes is influenced by where they find themselves along an inverted U-shaped affect arousal curve, known as the Yerkes-Dodson curve (McEwen and Lasley, 2002). When individuals experience low arousal levels, they may not have enough activation to engage System 2 into becoming alert and motivated to complete tasks at hand. Conversely, when individuals experience high arousal levels, they may find their attention and energy diverted from tasks to focus on the arousal itself—undermining System 2’s top-down control. As distress worsens, performance degrades steadily, eventually reaching a point of overwhelm or freeze. An individual’s neurobiological *window of tolerance* is the interval within which they are capable of regulating their arousal levels upwards or downwards, to remain alert but not so activated that they enter distress (Stanley, 2019). This moderate arousal zone is where System 2 processes are most effective, facilitating concentration, focus, and explicit memory formation, consolidation, and retrieval (Bremner, 2002; Sapolsky, 2004; Scaer, 2005; Porges, 2011).

**Figure 1:**
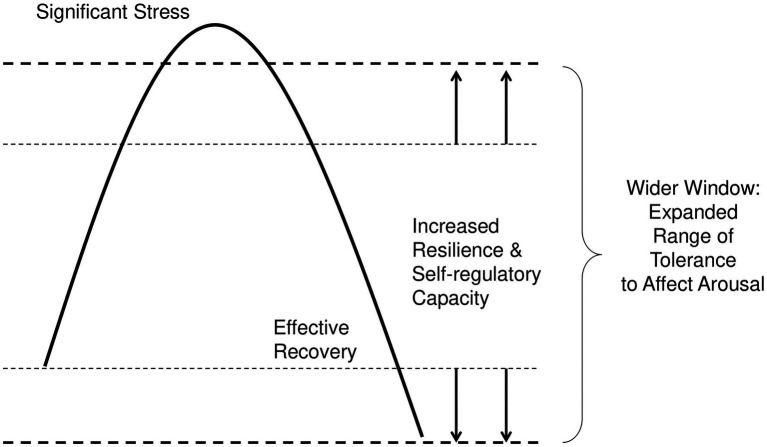
Widening the Window through Complete Recovery. This figure depicts how individuals can widen their neurobiological window of tolerance to affect arousal. When they experience affect arousal outside their comfort zone, followed by a complete recovery, they create a wider window. The wider their window, the greater their self-regulatory capacity, and the greater their capacity to integrate System 1 and System 2 processes during challenges.

Individuals inside their unique window of tolerance are more likely to engage in accurate neuroception and integrate System 1 and 2 processes successfully. Even during some affective arousal, they are able to regulate that arousal so as to keep System 2 processes fully online. (See Figure 1) Thus, they are more likely to perceive relevant internal and external cues; obtain and absorb adequate and appropriate information; and objectively assess and integrate that information. At any decision-point, they are more likely to search for all possible options; evaluate each option in terms of costs and benefits, by planning and considering its likely future effects; and then choose the strategically optimal decision best aligned with their values and goals (Stanley, 2018, 2019).

In contrast, when individuals experience stress or emotional arousal outside their window of tolerance, they are more likely to engage in *faulty* neuroception, such as neurocepting threat when a situation is truly neutral or safe, or neurocepting safety when a situation is truly dangerous (Porges, 2011; Stanley, 2019). Outside their window, individuals may also lose their capacity to use System 2 to modify or override System 1 assessments. In turn, stress and emotions are more likely to drive information search, assessment, and decision-making—resulting in impulsive and reactive behavior. Simultaneously, System 2 processes degrade. Indeed, System 2’s degradation at high arousal levels is one reason why memories of extremely stressful or traumatic experiences are often incomplete, contradictory, disordered, or fragmented (Bremner, 2002; Sapolsky, 2004; Scaer, 2005; Porges, 2011; Stanley, 2019).

As System 2’s top-down regulation worsens, individuals outside their window are also more likely to engage in maladaptive coping, such as alcohol/substance use and adrenaline-seeking, violent, or self-harming behaviors. Problematically, such coping techniques may then *further* degrade System 2’s regulatory capacity, lowering individuals’ inhibitions and increasing their likelihood of making unethical or reactive choices (Stanley, 2019; Stanley and Larsen, 2021). Thus, outside their window, an individual’s executive functioning, explicit memory, ability to relate effectively to others, and deliberate decision-making become degraded—showing just how important that window is in mediating the relationship between affect, cognition, and strategic decision-making.

**Figure 2:**
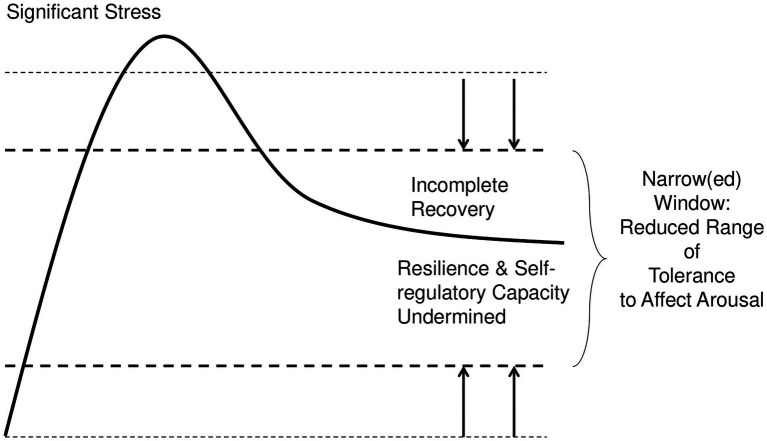
Narrowing the Window through Incomplete Recovery. This figure depicts how individuals can narrow their neurobiological window of tolerance to affect arousal. Their window can narrow *via* three pathways when they accumulate allostatic load without adequate recovery. The narrower their window, the more likely during challenges that System 2 processes will be operating in a degraded manner while System 1 processes drive decision-making and behavior.

What, then, determines the width of a leader’s unique window? Individual differences are initially wired through interactions between genetic traits and someone’s early caregiving environment. In addition, individual windows can narrow *via* three pathways over time: chronic stress or developmental trauma during childhood; shock trauma; and chronic stress or relational trauma during adulthood (Stanley, 2019; See Figure 2).

First, parents with insecure attachment styles and narrow(ed) windows are more likely to create the environmental conditions for their children to wire an insecure attachment style and develop a narrow window, too. When parents’ own capacity for regulating affect is compromised by narrow(ed) windows, it detrimentally influences their children in developing capacity for regulating affect as well (Ogden et al., 2006; Neigh et al., 2009; Siegel, 2012)—impairing the child’s ability to down-regulate stress and negative emotions during stressful experiences in the future. Indeed, substantial empirical research demonstrates how early life adversity can lead to long-term dysregulation and hypersensitization of several neurobiological systems reflective of a narrow window (for reviews, see Neigh et al., 2009; Stanley, 2019).

The second pathway to a narrow window is shock trauma. This occurs when individuals experience too much arousal too quickly. As an individual perceives helplessness, powerlessness, and loss of control, the acute emotional intensity and physiological arousal overwhelm their window—pushing them to the far-right end of the Yerkes-Dodson curve (Stanley, 2019). Importantly, shock trauma can occur with any large, abrupt change in arousal, whether that be a major event (e.g., mass shooting, terrorist attack, natural disaster, war) or a minor event (e.g., medical procedure, car accident). Such trauma is more common among those with already narrowed windows (Ogden et al., 2006; Stanley, 2019).

The third pathway to narrowing the window is chronic stress arousal (e.g., workaholism, tense relationships, chronic sleep deprivation, emotional labor) and “everyday” relational trauma (e.g., discrimination, harassment, abusive relationships). Without adequate recovery, these stressors induce low-level, long-term arousal that deplete the system, such that their cumulative effects build allostatic load (Stanley, 2019). Stressors on this pathway also include common culturally-sanctioned yet maladaptive coping strategies—compartmentalization, emotion suppression, the use of self-medicating substances, and adrenaline-seeking, addictive, violent, or self-harming behaviors (for reviews, see Stanley and Larsen, 2019, 2021)—that add to allostatic loads and narrow an individual’s window further.

These three pathways, alone or in combination, can narrow individuals’ windows—meaning that individuals may move outside their window because of multiple obvious *and nonobvious* arousal experiences. Regardless of pathway, the fundamental outcome is the same: as an individual’s allostatic load increases, they become more dysregulated and their window narrows (Stanley, 2019). The narrower their window, the more likely that System 1’s faulty neuroception—and stress arousal and emotions—drive the individual’s decisions, and the less likely they are able to access System 2’s deliberative cognitive decision-making capacities.

Thus, whether stress and emotions directly affect a leader’s strategic decision-making relies upon several factors: their current allostatic load; the current width of their window—and the self-regulatory capacity that window’s width allows; and their current affect arousal level (Stanley and Larsen, in press). When leaders’ arousal level pushes them outside their window, System 2’s executive functioning and cognitive capacity will be degraded; System 1’s stress and emotions will be more likely to drive their decisions; and integrative System 1/System 2 strategic decision-making will be corrupted. Leaders with particularly narrow windows may therefore have such compromised self-regulatory capacity that they are unable to make *any* decisions without being driven by their stress arousal, emotions, and impulses. On the other hand, leaders with particularly wide windows may be able to experience significant stressors, even shock trauma, but still be able to regulate their stress and emotions and execute System 2 processes effectively (Stanley and Larsen, in press).

In addition to windows directly affecting leaders’ own decision-making, the width of a leader’s window (and whether they are currently outside of it) also has indirect effects on their social environments—thereby affecting their groups’ *collective* window of tolerance. Leaders set the social and emotional tone for their group, often called the “command climate.” Affect contagion is an automatic, unconscious, and unintentional transference of affect; such contagion is most powerful in relationships that involve attachment bonds and/or power differences (Hatfield et al., 1994). Importantly, the relationship between a leader and their followers includes both characteristics, making them a likely source of affect contagion. Leader affect evokes similar physiological processes in followers, such that they automatically mimic their leader’s verbal and non-verbal cues and converge with their emotions (Neumann and Strack, 2000; Bono and Ilies, 2006; Johnson, 2008; Spoor and Kelly, 2009; Clarkson et al., 2020).

Leaders strongly influence how their subordinates will respond during stress, uncertainty, and change. Leaders can affect how their subordinates will interpret and make sense of stressful or challenging experiences (Weick, 1995; Bartone, 2006; Helms Mills et al., 2010; see also Cristofaro, 2021 for a review of how leaders can influence sensemaking in their organizations). At the same time, when leaders are perceived as competent, honest, trustworthy, and attuned to their subordinates’ physical, intellectual, emotional and social needs, they earn their subordinates’ trust and, by extension, boost their resilience (Catignani, 2004; Stanley, 2019).

The wider the leader’s window, the more likely that leader will be inside their window during stressful or challenging events. Leaders inside their windows are more likely to enact the constructive and cooperative relational strategies associated with secure attachment—to accurately perceive social and emotional cues from others, respect boundaries, communicate expectations clearly, speak honestly and openly, give and receive social support during stressful situations, and attune to the needs of their subordinates. These leader behaviors convey a calming and creative influence on their followers. In turn, through the principles of contagion, followers too feel comfortable exploring, learning, innovating, making mistakes, and growing. Followers learn that they can take risks, speak their minds, participate in group decisions, and confront difficulties with their leader’s support. In other words, the leader’s wide window helps endorse group behaviors that build collective resilience and widen the collective window (Stanley, 2019). With a wider collective window, the group is more likely to engage in accurate situational awareness, creative problem-solving, improvisation, adaptability, and connection with others. They are also less likely to experience affective arousal during interrupted plans or unexpected events (Weick, 1995; Stanley, 2019).

In contrast, a leader with a narrowed window is more likely to find themselves dysregulated outside their window. In this situation, the leader is likely to convey their affect arousal to their followers. The leader may convey hyper-aroused states (e.g., anger, fear) or hypo-aroused states (e.g., apathy, despair, victimhood, powerlessness), which are then ‘caught’ by followers. Such stress and emotion contagion increases the likelihood that all group members will resort to the insecure relational strategies associated with insecure attachment, including violence, conflict avoidance, gossip, defensiveness, disrespect, bullying, lying, bigotry, apathy, withdrawal, and indecision (Ogden et al., 2006; Siegel, 2012; Stanley, 2019). They are also more likely to engage in unethical and transgressive behavior (Stanley, 2019).

A dysregulated leader who is outside their window is also more likely to withdraw, limit information flow, involve fewer people in their decisions, and engage in micromanagement and other rigid control structures. These leader behaviors can increase uncertainty within the organization, fueling anxiety and exacerbating followers’ feelings of apathy and powerlessness (Stanley, 2018, 2019). These leader behaviors can also facilitate mistrust within the group, such that followers feel like they are competing with each other (Markman, 2021). In turn, coworkers are more likely to compete for the leader’s attention, take credit, and sow blame on others—fueling interpersonal tension within the group. These dynamics also undermine information sharing and cooperation, leaving the entire group less resilient and adaptable during change, uncertainty, or unexpected events. As a result, the leader’s dysregulated state can erode the group’s ability to cooperate, adapt, and learn—undermining followers’ resilience and narrowing the collective window (Stanley, 2019).

Of course, subordinates have their own windows of tolerance that predate interacting with and working for a well-regulated or dysregulated leader. However, because of the ripple effects of the leader’s window on the social environment, over time the leader’s window will begin to influence their followers’ individual windows, as well. For instance, the longer a wide-windowed subordinate works for a dysregulated leader who is outside their window, the more likely that subordinate will be subject to the toxic working conditions just described. In turn, that environment will create conditions for that subordinate to narrow their own window *via* the third pathway, through chronic stress arousal and relational trauma from repeated exposure to the environment the leader has created. Conversely, the longer a narrow-windowed subordinate works for a well-regulated leader who is inside their window, the more repeated experiences that subordinate will have of experiencing their leader enacting the relational strategies of secure attachment. Just as securely-attached parents set the conditions for their children to wire wide windows in childhood (Ogden et al., 2006; Siegel, 2012), leaders with wide windows can help their followers cultivate the traits that widen the followers’ individual windows, as well as the group’s collective window (Stanley, 2019).

## A Formal Illustration of Window Effects: Comparing Ardern’s and Trump’s COVID-19 Responses

We formally illustrate this argument using two leaders who represent the theory’s mechanisms. This illustration considers whether our novel theory’s expectations match reality, to establish whether there is a sufficient basis for a deeper causal analysis. Such plausibility probes are an important step between theory-building and larger-scale empirical assessment, particularly when theories are new and/or resource intensive to test (Eckstein, 1992; Kaarbo and Beasley, 1999). Specifically, we explore how the width of Prime Minister Ardern’s and President Trump’s respective windows directly affected their decision-making and indirectly affected their nations’ collective windows of tolerance during the early months of the COVID-19 pandemic.

We wanted our formal illustration to have contemporary policy relevance, which propelled our selection of the COVID-19 pandemic as the common decision-making environment. Then, we selected these two leaders for the pandemic illustration based on three factors. First, we sought to identify two countries for which the leaders shared a common first language (English), and for which there would be a sufficient number of news sources available in that language. Doing so ensured there would be enough data available to interpret sources consistently (without translation) and to illustrate the cases effectively; the United States and New Zealand satisfied this criterion. Second, we sought to identify two countries that held similar values and institutions, as a means of establishing that their leaders and societies would share reasonably similar decision-making environments. The United States and New Zealand have been directly compared in other studies for this reason; though their specific policies may differ, their pluralist “settler societies” share deeply held commitments to personal freedom, human rights, and the rule of law (Fischer, 2012, p. 32; see additional comparative examples in Baumann et al., 2019; Noack and Mahtani, 2019). Third, given how inconsistently the COVID-19 pandemic evolved around the world, we sought to identify two countries for which the timeline of first documented cases and governmental action were approximately equivalent. The first cases documented in the United States and New Zealand fell between January and February of 2020, and both countries took their first major governmental lockdown actions within a week of each other in March 2020 (World Health Organization, 2021a,b).

After establishing that the United States and New Zealand satisfied all three criteria, we collected information for both illustrative cases *via* an intensive search of available primary sources (including interview and speech transcripts, as well as subjects’ personal social media statements) and secondary sources (including biographies and accounts published in reputable news sources, often based on interviews with the subjects’ closest associates). We extracted data from this narrative review when it included (1) evidence of Ardern’s/Trump’s window wiring and/or width throughout their lifespan, and (2) evidence of Ardern’s/Trump’s decision-making during the first year of the COVID-19 pandemic. In line with the motivations behind a plausibility probe, information was included whether or not it satisfied the window theory’s expectations; full case narratives were written that included all collected information. Upon review, we determined those cases showed no disconfirming evidence, but that some of the confirming evidence was repetitive. For the sake of clarity, we therefore excluded some of the duplicative confirming evidence. The remaining evidence follows in the illustrations below.

### Prime Minister Jacinda Ardern’s Relatively Wide Window

Evidence from Ardern’s life suggests that she developed a relatively wide window throughout her lifespan. In line with the first pathway, Ardern’s primary caregivers appear to have had a strong, loving relationship and been well-regulated themselves. Ardern describes her school cafeteria worker mother as “the epitome of kindness” (Blackwell, 2020, p. 32), who “instilled in her and her sister a strong sense of service” (Lester, 2019, para. 5). Until she was eight, Ardern lived in the poor, predominantly Maori, forestry town of Murupara, known for its crime, poverty, and addiction. While she was “relatively insulated from the injustice around her” (Chapman, 2020, p. 2; see also Duff, 2019), Ardern observed her police officer father deescalate many altercations—something she credits for teaching her interpersonal diplomacy and negotiation skills (Chapman, 2020). The Mormon family consumed no alcohol, caffeine, or tobacco (Duff, 2019; Chapman, 2020), and though Ardern left the Mormon Church later, she praises her upbringing for helping her develop a sense of optimism, service, and responsibility (Roy, 2018).

After moving to Morrinsville, 12-year-old Ardern was elected student council president, where she discovered she “was very good at being in charge” and “had a spontaneous and genuine interest in advocacy” (Chapman, 2020, p. 3). She served as a prize-winning debater, the sole elected student representative to the school’s Board of Trustees, and leader of the local Students Against Drunk Driving chapter (Chapman, 2020). Ardern’s parents have told reporters they always thought she could be prime minister someday; as her mother said, “She was mature beyond her years and had incredible common sense” (quoted in Duff, 2019, p. 41). These childhood experiences suggest through her early social environment, Ardern likely developed a secure attachment style and secure relational strategies, allowing her to wire a wide window.

Ardern would later experience a shock trauma event during her tenure as prime minister—though her response, per the second pathway, provides further evidence of a wide window. On March 15, 2019, mass shootings at two mosques in Christchurch killed 51 people; it was New Zealand’s deadliest shooting in modern history, and its first since 1997 (Fattal, 2019). Although in shock, Ardern’s immediate response was widely recognized for its decisiveness and compassion (Duff, 2019; Lester, 2019; Chapman, 2020). She addressed the nation several times, used inclusive language, wore a head-scarf, and met with Christchurch survivors and community leaders (Duff, 2019; Lester, 2019; Chapman, 2020). As one journalist explained, “she listened to people who were grieving and reacted with kindness. Her actions dismantled the notion that leaders have to be emotionless and uncaring to retain authority” (Duff, 2019, pp. 146–147). Within the week, her government enacted sweeping changes to New Zealand’s gun laws by banning all assault rifles and military-style automatic weapons (Lester, 2019). She also hosted an international summit to bring world leaders and technology companies together to ratify the “Christchurch Call”—a global pledge to keep internet platforms from being used to spread hate (Duff, 2019; Mahtani and Fifield, 2019; Chapman, 2020).

Per the third pathway, Ardern has experienced chronic stress and relational trauma, but again shows evidence of a wide window. She became a Labor member of parliament (MP) at just 28, then twice ran against (and lost to) National’s Nikki Kaye for an elecorate seat—which the media widely reported on using sexist terms, such as the “Battle of the Babes” (Duff, 2019, p. 102; Chapman, 2020). In 2017, Arden became an electorate MP and rose to the rank of Labor Party deputy leader. When the party leader stepped down seven weeks before the election, she became party leader and then prime minister, garnering even more media sexism: News shows described her as a “pretty little thing” who would “look good” as prime minister, while commentators painted her as vacuous and superficial, asking if she “really has what it takes” and calling her Labor’s “show pony” (Duff, 2019, pp. 109–114; see also Chapman, 2020).

Ardern learned she was pregnant during her negotiations to form a coalition government, adding another chronic stressor to her load (Duff, 2019; Chapman, 2020). She “carried on as normal, forming a government and doing her best to not let world leaders see that she was trying not to throw up while speaking with them” (Chapman, 2020, p. 110). Ardern became the second world leader to give birth while in office; the first to take maternity leave; and the first to bring her breastfeeding infant to the United Nations General Assembly (Duff, 2019; Chapman, 2020).

Ardern may have mitigated any potential window-narrowing effects of these stressors through her strong social support; consummate relationship-building skills; and unprecedented openness and connection with her followers. First, she reports a strong relationship with her romantic partner Clarke Gayford, noting “I can only do everything because I have help, by which I mean Clarke” (quoted in Chapman, 2020, p. 131). Second, throughout her career, Ardern has been a “master networker” (Chapman, 2020, p. 27); it has been said that “building relationships has always been Ardern’s greatest strength” (Duff, 2019, p. 110). This provided her a large web of friends and allies, and helped her establish a coalition government (Chapman, 2020). Third, since 2008, Ardern has connected directly with her followers, using Facebook Live and social media to allow voters to have their questions answered immediately (Chapman, 2020). On her way to being sworn in as prime minister, she told Radio New Zealand that she wanted citizens “to feel that [the government is] open, that it’s listening, and that it’s going to bring kindness back” (quoted in Duff, 2019, p. 156). In all her communications and media interviews, she demonstrates “genuine empathy and a sense of humor” (Chapman, 2020, p. 91) and presents herself as authentic, “self-deprecating and down to earth” (Duff, 2019, p. 187)—even making her first policy pronouncement as prime minister *via* Facebook Live from her home, while on maternity leave (Duff, 2019).

### Inside the Window: Ardern’s and New Zealand’s Response to COVID-19

Ardern’s decision-making during the early stages of the COVID-19 pandemic provides a particularly apt illustration of her wide window. While she earned some criticism for choices regarding vaccine rollout (BBC, 2020; Hollingsworth, 2021), Ardern’s wide window enabled her to make effective decisions and to influence the entire nation’s successful pandemic response.

Reporting indicates Ardern was able to access System 2 executive functioning and cognitive processes, regulate her stress and emotions, make effective decisions, and update her decisions as new information arose. She actively sought out and incorporated input from epidemiologists, independent experts, and business and community leaders, all to ensure her government had the best science and health advice (Wilson, 2020). In a November 2020 survey, three quarters of New Zealand scientists believed their policymakers were taking scientific evidence into account during the pandemic—the highest national approval among more than 25,000 scientists surveyed worldwide (Rijs and Fenter, 2020). After making Christchurch anniversary event plans in line with existing scientific evidence, then receiving new information, Ardern immediately cancelled the event and instituted self-isolation policies (BBC, 2020). All of these decisions provide evidence of her wide window allowing for successful integration of Systems 1 and 2; in contrast, there are thus far no reported examples of stress or emotions detrimentally influencing Ardern’s pandemic decision-making.

In line with the theory’s expectations, Ardern’s wide window also appears to have shaped New Zealand’s collective pandemic performance. Ardern appealed directly to the public with simple, consistent, and emotionally intelligent messaging. During her regular Facebook Live chats, she used easily understood language to translate risk and uncertainty (Friedman, 2020; Wilson, 2020)—offering citizens a clear view of the future and the stakes involved, rather than minimizing the virus’ true threat (Radio New Zealand, 2020; Wilson, 2020). She appeared jointly with the Director-General of Health to promote apolitical public health guidance, and introduced a straightforward four-level alert system to help the public understand when, how, and why the government would implement policy responses (Chapman, 2020; Wilson, 2020).

At the same time, her messaging was emotionally intelligent. She deliberately repeated her simple and encouraging catchphrases during all public communications: “Go hard, go early. Stay in your bubble. Team of five million. Be strong but be kind” (Chapman, 2020, p. 207). She used identifiable examples, such as telling fellow parents that she understood how hard it would be to avoid playgrounds (Friedman, 2020), and humor, declaring the tooth fairy and Easter bunny ‘essential workers’ who could still visit families (Chapman, 2020). As one biographer notes, Ardern does not “use fear to motivate; instead, her weapon was inclusivity” (Duff, 2019, p. 155). Promoting kindness and empathy, she emphasized that “we are all now putting each other first. And that is what we as a nation do so well” (quoted in Dada et al., 2021, p. 7). Ardern also encouraged citizens to make phone trees to check on each other (Mayer and May, 2021) and included resources about kindness on the COVID-19 governmental website (Wilson, 2020). As one scholar noted, Ardern sought to “use the bully pulpit to cue society toward [their] better angels” (quoted in Friedman, 2020, para. 11).

The successful impact of Ardern’s wide window on her decision-making and New Zealand’s collective window manifested in three ways. First, in October 2020, Ardern’s Labour Party was re-elected in a landslide that allowed for a single-party government, the first time since 1996 (CNBC, 2020)—a “historic shift” that was “one of the biggest swings in New Zealand’s electoral history” (Menon, 2020, para. 3). Second, leaders who might have otherwise opposed Ardern’s policies bought into them and amplified her message further: Opposition leaders urged residents to follow officials’ recommendations, mayors of regions experiencing repeated lockdowns adopted her messaging, and businesses around the country encouraged customers to stay positive and “be kind, stay safe” (Blackwell, 2020, p. 15; see also BBC, 2020). Third, New Zealand has been globally recognized as a pandemic success story. To date, New Zealand has lost 27 lives to COVID-19, with an average daily case rate below 20 (World Health Organization, 2021a). When rare cases have reappeared, New Zealanders readopt quarantine measures and once again adhere to guidelines (Perry, 2020, 2021). There have been no major protests of New Zealand’s COVID-19 policies (Chapman, 2020), and by early 2021, seven of ten New Zealanders said they thought the country was “heading in the right direction” (Roy Morgan Research, 2021).

### President Donald Trump’s Relatively Narrow Window

In contrast to Ardern, evidence from Trump’s lifespan suggests that he built allostatic load and narrowed his window. During Trump’s presidency, a group of clinical psychologists and psychiatrists at a Yale symposium argued it was their moral and civic “duty to warn” the public about Trump’s psychopathology—a duty which they argued supersedes the American Psychological Association’s “Goldwater rule,” inhibiting mental health professionals from diagnosing public figures they have not personally examined (Lee, 2019). Drawing on evidence in the public record, contributors argued that Trump exhibits symptoms of malignant narcissism, present hedonism, compulsive impulsivity, and attention deficit/hyperactivity disorder, as well as early signs of dementia or Alzheimer’s. The analysis here builds on this earlier debate, providing evidence from Trump’s lifespan to explore how he narrowed his window over time.

In line with the first pathway, Trump’s mother Mary was reported by his niece—a clinical psychologist—to be “the kind of mother who used her children to comfort herself rather than comforting them. She attended to them when it was convenient for her, not when they needed her. Often unstable and needy, … she frequently put herself first” (Trump, 2020, p. 23). When Trump was a toddler, Mary had multiple emergency surgeries and hospital stays, and Trump’s father Fred became the default primary caregiver. Fred was a “high-functioning sociopath” and workaholic, who focused almost completely on business and exhibited “a lack of empathy, a facility for lying, an indifference to right and wrong, [and] abusive behavior” (Trump, 2020, p. 24). Fred rebuffed his children’s desire for soothing and care; as a result, for Trump and his younger brother, “‘needing’ became equated with humiliation, despair, and hopelessness” (Trump, 2020, p. 25).

Parents’ lack of emotional and physical availability may wire insecure avoidant attachment styles in their children, creating chronic stress arousal and disconnections between the child’s inward states and outward behaviors (Ogden et al., 2006; Siegel, 2012; Stanley, 2019). This may have been the case for Trump, as his niece highlights that he “began to develop powerful but primitive defenses, marked by an increasing hostility to others and a seeming indifference to his mother’s absence and his father’s neglect” (Trump, 2020, p. 27). Accounts of Trump’s childhood behavior exhibit many other signs of avoidant attachment as well, including difficulty reading social cues, tormenting his younger brother, bullying other children, arguing with teachers, and eventually being kicked out of his private school and sent to a military academy “as a way to rein him in” (Trump, 2020, pp. 43–49). These experiences of early childhood abandonment, neglect, abuse, and chronic stress arousal likely narrowed Trump’s window, with lifelong implications.

Trump also endured a shock trauma event during adulthood that may have further narrowed his window *via* the second pathway: the early death of his older brother Freddy. Although their father’s demanding, unyielding, and highly competitive streak was “doubled in Donald” (Kirk and Wiser, 2017), Freddy turned instead to alcoholism under the stress of Fred’s criticism, humiliation, and preferential treatment of Trump—and died of a heart attack at 42 (Trump, 2020). Trump had a contentious relationship with Freddy, often scolding him for his behaviors, but later lamented that he had not understood Freddy’s struggles (Horowitz, 2016).

Per the third pathway, Trump’s lifestyle exhibits chronic stress arousal. A self-described workaholic, Trump claims that for many years he has only slept 3–5h per night, a habit he developed in business (United States Office of the Press Secretary, 2018; Le, 2019). As a result, he exhibits many symptoms of chronic sleep deprivation, including impulsivity, poor concentration, difficulty processing information, and difficulty regulating emotions (Egan, 2016; Devega, 2018). Trump also drinks “upward of twelve Diet Cokes a day” and “has a horrible diet and does not exercise,” preventing recovery and likely exacerbating his everyday allostatic load (Trump, 2020, p. 13). Also reflecting and exacerbating his narrowed window, Trump had a documented history of maladaptive coping, including inappropriate, adrenaline-seeking, and even violent behavior. In addition to alleged serial philandering, since 1995 Trump has been publicly accused of rape, sexual assault, sexual harassment, or inappropriate touching by 16 women, many with multiple contacts corroborating their allegations (Kelly, 2017; Trump, 2020).

Evidence of impulsive and reactive behavior continued during his presidency. The Trump Administration, which saw record-breaking departures of Cabinet-level officials, was reportedly plagued by “paranoia, insecurity and scheming—and of an inner circle gripped by fear of Trump’s spasms” (Rucker and Costa, 2019, para. 7). As one senior administration official wrote, “meetings with him veer off topic and off the rails, he engages in repetitive rants, and his impulsiveness results in half-baked, ill-informed and occasionally reckless decisions that have to be walked back” (Taylor, 2018, para. 14). This is further reflected in his presidential Twitter use, wherein many tweets appeared to be almost-instantaneous responses to programs that Trump was watching; one journalist determined that Trump took, on average, six minutes to compose and post a tweet after seeing topics covered on television (Altman, 2019). As his niece characterized it, “Donald today is much as he was at 3years old: incapable of growing, learning, or evolving, unable to regulate his emotions, moderate his responses, or take in and synthesize information” (Trump, 2020, p. 197).

### Outside the Window: Trump’s and the United States’ Response to COVID-19

Trump’s pandemic decision-making offers a clear illustration of the impact of his narrow window. Some might suggest that Trump’s approach of claiming credit, avoiding blame, sowing division, and fostering anti-government messaging was a ‘rationalist’ strategy based on what had made him successful in the past (Kapucu and Moynihan, 2021). Though that strategy’s behaviors and the behaviors of a narrow window are admittedly very similar, the fact that Trump kept “instinctually returning” to that strategy, even as it undermined his failed 2020 reelection effort, suggests this ‘rationalist’ approach may not completely explain his pandemic behavior (Kapucu and Moynihan, 2021, p.10). Instead, the consistency between the window theory’s expectations and the observed realities suggest it is a powerful alternative explanation for his decision-making.

The pandemic began in earnest during Trump’s first impeachment trial, which threatened to remove him from power after several years of high-profile criticism of his leadership. Trump may have neurocepted this political environment as threatening, which—in concert with an already narrowed window—implies his self-regulatory capacity was likely diminished when COVID-19 decision-making started. For instance, although the Central Intelligence Agency had warned about a potential pandemic from China in November 2019 (Rutledge, 2020)—and experts across the intelligence and public health communities agreed by January 2020 (Lipton et al., 2020)—Trump told aides at the start to “stop panicking” and that he suspected ‘Deep State’ actors within the administration were trying to mislead him pre-election (Lipton et al., 2020, para. 88; see also Kapucu and Moynihan, 2021).

Evidence of the impact of this narrow window on his pandemic decision-making abounds. First, Trump inconsistently considered scientific advice and often rejected alternative viewpoints—reflecting the narrowed attentional focus, limited information flow, and rigid control structures of a leader outside their window (Stanley, 2018, 2019). Although Trump eventually allowed the convening of a White House coronavirus task force, his “inability or unwillingness to absorb warnings coming at him” meant that when that task force announced severe virus mitigation recommendations, Trump lashed out at the team for scaring people unnecessarily and replaced the task force leadership (Lipton et al., 2020, para. 35). Trump also sidelined several other pandemic officials, including the agency leader responsible for developing COVID-19 vaccines, after they said Trump’s claims “lack scientific merit” (quoted in Shear and Haberman, 2020, para. 4). Trump further illustrated this rigid commitment to his narrow perception by emphasizing that he was “inclined not to speak with anyone who is insufficiently appreciative of his administration’s efforts” (Olorunnipa et al., 2020, para. 9).

Second, dysregulated emotions, especially impatience, appeared to drive many of Trump’s pandemic decisions. After Trump finally announced a nationwide lockdown, he told Congress to “just stay calm, and it will go away” and informed Americans the country would open by Easter (Rutledge, 2020, p. 507). By mid-April, Trump grew publicly impatient with the recommendations he had grudgingly endorsed; instead, he and his team “convinced themselves that the outbreak was fading, that they had given state governments all they needed to contain its remaining ‘embers,’ and that it was time to ease up on the lockdown” (Shear et al., 2020, para. 4). Accordingly, daily briefings with Trump ended in late April, and the task force barred infectious disease specialist Dr. Anthony Fauci from making television appearances, “lest he go off message and suggest continued high risk from the virus” (Shear et al., 2020, para. 49). However, when reports the task force *itself* would end in May provoked outrage, Trump changed course and insisted its daily meetings would not end (Lipton et al., 2020; Shear et al., 2020).

Trump’s narrowed window also indirectly shaped the United States’ collective pandemic performance in three ways. First, Trump engaged in erratic, inconsistent, factually untrue, and often impulsive messaging, which stoked public uncertainty, anxiety, and confusion. In January 2020, Trump quickly downplayed the threat on television (Lipton et al., 2020; Rutledge, 2020); then, when it became clear a lockdown was needed, Trump praised emergency workers, encouraged social distancing, and discouraged large gatherings. However, “he also, at different times, more forcefully promoted the opposite of these messages,” such that “his staff ultimately decided they were doing more harm than good” (Kapucu and Moynihan, 2021, p. 10). Indeed, Smith (2020) notes that Trump at times derided masks as “politically correct” (para. 28), then suggested they were “patriotic” (para. 1). Likewise, he said schools needed to reopen or risk losing funding (Baker et al., 2020), then said this would not apply for all schools (Binkley, 2020). Against scientific consensus, Trump offered varied, off-the-cuff remarks about hydroxychloroquine, remdesivir, and other ‘remedies’ such as ultraviolet light and disinfectant injections (Broad and Levin, 2020; Niburski and Niburski, 2020). White House officials acknowledged that these impromptu statements were neither scripted nor in line with policy positions (Lee et al., 2020).

Second, Trump’s impatience created a “leadership vacuum” that undermined state and local leaders’ attempts to respond successfully to the virus (Shear et al., 2020, para. 17). While many state officials warned the pandemic was far from under control, Trump agitated to lift the lockdown and pushed states to reopen their economies. He “began criticizing Democratic governors who did not ‘liberate’ their states” (Shear et al., 2020, para. 10), transmitting hyperarousal to his followers *via* Twitter and media appearances by enthusiastically encouraging protests at state capitals and calling state leaders dictators (Shear et al., 2020). One particularly radical result of this contagion was the planned kidnapping of Michigan Governor Gretchen Whitmer, in what investigators say was a plot by anti-government extremists who were angry over her “tyrant” coronavirus policies (Snell and Burke, 2020, para. 2).

Third, Trump’s dysregulated leadership and erratic messaging disrupted the decisions of his inner circle. His principal aides followed his tendency to reject alternative perspectives, by adopting “a similar strategy of issuing threats or isolating their rivals, undermining efforts to manage the outbreak” (Diamond, 2021, para. 18). At various points, White House Chief of Staff Mark Meadows, Trump’s son-in-law Jared Kushner, the Vice-President’s Chief of Staff Mark Short, and others were reported as yelling, complaining, and exploding in anger at other officials—with reporters indicating that the White House had become “a toxic environment in which no matter where you turned, someone was ready to rip your head off or threatening to fire you” (quoted in Diamond, 2021, para. 23). The discord sowed by Trump’s inconsistencies, rejections, and erraticism meant that beyond his own dysregulated decision-making, “no one was in charge of the [pandemic] response… there was no accountability, and the response was rudderless” (Diamond, 2021, para. 30).

The negative impact of Trump’s window on his own decision-making—and on the United States’ collective approach—manifested in several ways. First, public anxiety, confusion, and distrust became widespread. By August 2020, 58% percent of Americans reported being confused by the US government’s messages; 46% reported believing social order had worsened; and 47% reported expecting a second lockdown (State Policy Network, 2020). In October 2020, two-thirds of American adults said they were worried they or someone in their family would get sick from COVID-19, while 55% said they thought Trump was intervening in the Federal Drug Administration’s scientific process of reviewing and approving a vaccine (Kaiser Family Foundation, 2020). Second, Trump lost re-election, as exit polls showed Americans who viewed the pandemic as the most pressing issue facing the country favored his opponent Joseph Biden (Medina and Russonello, 2020). Third, and perhaps most importantly, the United States’ pandemic response was widely deemed a failure. The country has suffered more than 663,000 COVID-19 deaths, with a continued daily case rate of more than 156,000 (World Health Organization, 2021b).

## Discussion: Developing Testable Hypotheses

This preliminary illustration underscores the potential impact of leaders’ windows of tolerance on their decision-making, and future research should prioritize testing a number of hypotheses to causally evaluate the theory’s validity. We present two sets of hypotheses to be tested. The first are *direct hypotheses*, specific predictions about the direct effects of leaders’ windows on their own decision-making. The first direct hypothesis posits that leaders who have accumulated allostatic load *via* any of the three pathways explored herein are likely to have narrower windows of tolerance than those who have not accumulated such load—and thus, will have diminished capacities to engage in strategically optimal System 2 decision-making.

The second direct hypothesis posits that all leaders who face acute crises may initially experience affect arousal that extends beyond their window, but leaders with relatively wide windows will have the self-regulatory capacity to down-regulate that arousal and access System 2 decision-making. The wider the leader’s window, the more quickly they should be able to regulate arousal and access optimal decision-making. In contrast, leaders with narrow(ed) windows may be unable to down-regulate their affect arousal—and therefore may find System 2 processes degraded, and stress and emotions driving their decisions, for the crisis duration.

The third direct hypothesis is that all leaders facing prolonged conflicts will experience some narrowing of their window, but leaders with wide windows will have greater self-regulatory reserves to guard against such depletion and protect their System 2 processes. In contrast, leaders with narrow(ed) windows will have fewer regulatory reserves and, as such, may remain outside their window for the duration of the prolonged stressful event, with concomitant detrimental effects on their decision-making.

In addition to these direct hypotheses regarding the window’s impact on leader decision-making, we also present two *indirect hypotheses* that predict how leaders’ windows shape the collective decision-making of their groups. According to the first indirect hypothesis, leaders who are inside their windows will transmit a calming, adaptive, and balanced decision-making process to their followers. Specifically, when leaders are inside their window and effectively integrating Systems 1 and 2 on a regular basis, their followers will cognitively and affectively appraise that they can take risks, speak their minds, confront difficulties, and engage in creative problem-solving. In contrast, leaders who are outside their window are likely to convey their stress arousal and negative emotions to their followers, such that the resulting contagion will push followers to insecure and defensive relational strategies that impede the group’s ability to effectively cooperate, adapt, and learn.

The second indirect hypothesis builds on recent theorizing integrating the interrelationships of affect, cognition, and sensemaking within groups. Sensemaking occurs when “the current state of the world is perceived to be different from the expected state of the world” (Weick et al., 2005, p. 409). Recent research shows how sensemaking is both driven by body-based affective states and subject to cognitive and affective contagion—with such contagion moderated by leaders’ sensegiving influence (for a review, see Cristofaro, 2021). Furthermore, Weick suggests that the more rigid and well-organized an individual’s and/or an organization’s expectations and response sequences are, the greater affective arousal they will experience at having those expectations and response sequences interrupted, in turn stimulating the sensemaking process (Weick, 1995).

With this context in mind, the second indirect hypothesis is that leaders who are inside their windows should experience more capacity for flexibility and improvisation when their expectations and response sequences are interrupted. They are less likely to perceive such interruptions as threatening and, in response, less likely to experience high levels of physiological and/or emotional arousal. In turn, they are more likely to convey a calming and creative influence on their followers’ sensemaking, as well. In contrast, leaders who are outside their windows are more likely to experience high affect arousal levels at having their expectations and response sequences interrupted. They are more likely to experience difficulty in identifying other courses of action when their preferred, expected course has been thwarted. In turn, their higher arousal levels are more likely to shape their own sensemaking and—*via* contagion during sensegiving to their followers—shape their followers’ sensemaking, as well. These hypotheses offer the conceptual guidelines to effectively test the window theory, but there are also several practical considerations for future research. First, studies should consider a diversity of decision-making environments for testing the theory. The argument put forth here draws on large-scale political decision-making environments that are particularly effective incubators for affect and decision-making—but evaluating the theory’s consistency across the upper echelons of smaller-scale corporate decision-making environments is essential for establishing its reliability. Second, future research should also seek to examine multiple leaders across multiple time periods, as a means of establishing the consistency and durability of the window theory’s key hypotheses. Third, prospective tests of the window theory should prioritize both quantity and quality of primary source material for their assessments. Establishing detailed life histories, symptoms, and coping styles of leaders is essential to accurately evaluating the width of their windows and ensuring the findings are not overly deterministic.

Finally, future research must develop effective tests for establishing the comparative influence of the window and leaders’ self-regulatory capacity against other factors that influence decision-making. For instance, classic studies comparing the structural differences of democratic systems find that the separation of powers in presidential systems leads to greater divisions in authority than the relatively centralized decision-making processes of parliamentary systems, which may translate into presidential systems experiencing a greater breakdown in decision-making, fewer commitment problems, and more durable decisions—though evidence for both systems is mixed (e.g., Lijphart, 1992; Moe and Caldwell, 1994; Tsebelis, 1995). At the same time, research exploring individual-level traits finds that gender influences the types of policies that leaders support, leaders’ openness to policy change, and their inclusivity in decision-making (e.g., Weikart et al., 2007; Swers, 2020). Integrating these and other factors into any future window theory research will be essential to assessing the relative explanatory leverage of the window theory against alternative arguments. When these recommendations are applied in concert with the theory and hypotheses herein, they will ensure that the ‘affect revolution’ continues to nurture our understanding of strategic decision-making in a range of environments.

## Conclusion

Theories about the relationship between affect, cognition, and political decision-making have made many advances, but have neglected affect arousal, allostatic load, and self-regulatory capacity in their efforts to explain decision outcomes. As we explore, this under-theorization largely results from their failure to consider when the deliberate cognitive strategies of System 2 decision-making may be inaccessible. Furthermore, existing theories fall short in connecting the dots between leaders’ own self-regulatory capacity and its ripple effects onto their groups’ decision-making and behavior. In response, we have introduced the neurobiological window of tolerance and leaders’ self-regulatory capacity to clarify when affect might drive decisions for one leader but not for another.

We formally illustrated the theory’s mechanisms by comparing Prime Minister Ardern’s and President Trump’s responses to the coronavirus pandemic in their respective nations. Ardern’s relatively wide window not only directly shaped her own effective decision-making, but also indirectly affected New Zealand’s successful collective response. In contrast, Trump’s relatively narrow window allowed stress arousal and emotions to drive much of his decision-making, while also indirectly undermining the United States’ collective response.

As noted above, using the COVID-19 pandemic to illustrate these mechanisms has some limitations—despite the important policy relevance of this example. Beyond the two nations’ fundamental geographic differences (e.g., land mass and international borders) and demographic differences (e.g., population size and urban density), other factors may have influenced their respective pandemic outcomes, above and beyond the influence of the leaders’ windows of tolerance. For instance, although COVID-19 vaccines were available in the United States roughly 2 months earlier than in New Zealand, the United States encountered more anti-vaccination sentiment, disinformation, and media distrust than New Zealand (Baumann et al., 2019; Myllylahti and Treadwell, 2021). The two countries’ differing healthcare system enrollments—universal healthcare in New Zealand, and a private/public insurance system in the United States—may also have lowered Americans’ willingness to engage with medical services for COVID-19 vaccination or treatment (Kelly et al., 2021). Furthermore, while political polarization has increased in both countries, this increase in the United States is at least three times larger than that in New Zealand—which may have influenced their respective citizens’ likelihood of following government guidelines (Boxell et al., 2020).

Despite these limitations, however, this illustration offers insight into the potential explanatory power of the window theory and its application to political leadership. While the previous section outlined theoretical implications and questions for future research, important policy implications also flow from this illustration. For instance, as this illustration shows, leaders who take active steps to keep their own window wide will have more capacity for effective decision-making during crises. At the same time, leaders who prioritize self-care to stay regulated themselves—such as through getting enough sleep and cardiovascular exercise—signal to their followers that self-regulation is a critical aspect of successful performance. As a result, the entire group may come to prioritize self-regulatory behavior, which can widen the collective window. Furthermore, understanding leaders’ windows and their self-regulatory capacity could (and should) aid in political leader selection in the first place, since leaders’ self-regulatory capacity can play a significant role in shaping the policies that govern citizens’ lives. Indeed, as the COVID-19 pandemic shows, political leaders’ self-regulatory capacity might actually influence who lives and who dies in a country’s crisis response.

## Data Availability Statement

The original contributions presented in the study are included in the article, further inquiries can be directed to the corresponding author.

## Author Contributions

KL researched and initially drafted the literature review and COVID-19 illustration sections. ES researched and initially drafted the Ardern and Trump leader window sections, as well as the theory and hypotheses sections. All authors were involved in assembling and revising the complete manuscript.

## Conflict of Interest

The authors declare that the research was conducted in the absence of any commercial or financial relationships that could be construed as a potential conflict of interest.

## Publisher’s Note

All claims expressed in this article are solely those of the authors and do not necessarily represent those of their affiliated organizations, or those of the publisher, the editors and the reviewers. Any product that may be evaluated in this article, or claim that may be made by its manufacturer, is not guaranteed or endorsed by the publisher.
